# Bioelectronic interfaces by spontaneously organized peptides on 2D atomic single layer materials

**DOI:** 10.1038/srep33778

**Published:** 2016-09-22

**Authors:** Yuhei Hayamizu, Christopher R. So, Sefa Dag, Tamon S. Page, David Starkebaum, Mehmet Sarikaya

**Affiliations:** 1 GEMSEC, Genetically Engineered Materials Science and Engineering Center, Materials Science and Engineering, University of Washington, Seattle, WA 98195, USA; 2 Materials Science and Engineering, Tokyo Institute of Technology, Tokyo 152-8550, Japan; 3 PRESTO, Japan Science and Technology Agency (JST), 4-1-8 Honcho, Kawaguchi, Saitama 332-0012, Japan

## Abstract

Self-assembly of biological molecules on solid materials is central to the “bottom-up” approach to directly integrate biology with electronics. Inspired by biology, exquisite biomolecular nanoarchitectures have been formed on solid surfaces. We demonstrate that a combinatorially-selected dodecapeptide and its variants self-assemble into peptide nanowires on two-dimensional nanosheets, single-layer graphene and MoS_2_. The abrupt boundaries of nanowires create electronic junctions *via* spatial biomolecular doping of graphene and manifest themselves as a self-assembled electronic network. Furthermore, designed peptides form nanowires on single-layer MoS_2_ modifying both its electric conductivity and photoluminescence. The biomolecular doping of nanosheets defined by peptide nanostructures may represent the crucial first step in integrating biology with nano-electronics towards realizing fully self-assembled bionanoelectronic devices.

Interface between electronic materials and biomolecules is essential in integrating human-made devices with biological systems^1^,^2^. Inspired by biology, DNA^3^ and proteins^4^ have been engineered to spontaneously form exquisite molecular architectures on solid surfaces. However, the integration of the biomolecules at solid interfaces still needs to be understood from the standpoint of bridging biology with electronic devices^5^. Due to their atomically flat surfaces and rich electronic properties, atomic single layer materials represented by graphene^6^ offer an ideal platform to study electronic coupling of biomolecules with solid surfaces. Self-organized structures of peptides^7^,^8^,^9^ may be utilized for direct formation of bioelectronic interfaces with controlled electronic states of single layer materials. Here, we demonstrate that designed dodecapeptides spontaneously form nanostructures templated by 2-dimensional (2D) layered materials, including graphite, MoS_2_, WSe_2_, WS_2_, MoSe_2_, and hBN. We show that peptides assemble into organized or disorganized structures distinctively modifying electrical and optical properties of single-layer graphene and MoS_2_, respectively. The ability to regulate electronic states of atomic single layer materials defined by peptide sequences establishes the first step in a unique route to integrate biology with 2D nanomaterials towards realizing fully self-organized biomolecular-electronics.

Proteins regulate biomineral formation in organisms through specific molecular interactions leading to self-assembly of hard tissues (bones, teeth, shells, spicules and particles) with exceptional physical functions^10^,^11^. Learning from nature, solid-binding peptides^12^,^13^,^14^ have recently been developed and became ubiquitous molecular building blocks in nanotechnology^15^ and medicine^16^. Besides their enzymatic function in cell-free biomineralization and utility as molecular linkers bridging multiple biomolecules and materials, some solid-binding peptides also self-assemble into highly ordered structures on surfaces of Au (111)^9^ and cleaved graphite^17^. … “Natural peptides, *e.g*., amyloids, also form ordered patterns on mica and graphite^7^,^8^. These peptide nanostructures exhibit the same symmetry as the underlying crystal structure, indicating that the peptide assembly is templated by the solid surface. Single-layer graphene has also been utilized to demonstrate peptide assembly^18^, and electrical interactions of peptides with graphene have been investigated^19^; however, in this case, the surface assembly of peptides does not display ordered structures. Despite the progress in peptide assembly on surfaces of 2D materials, direct formation of highly ordered monolayer peptide nanostructures on single-layer 2D nanomaterials and their effect on physical properties has not yet been examined. Graphene is the most studied 2D single-layer material due to its simple structure and rich electric properties^6^. Transition metal dichalcogenides (MoS_2_, WSe_2_, *etc.*) are new members of 2D semiconductors with unique electronic^20^,^21^ and optical properties^22^,^23^,^24^. Designing and controlling the peptide assembly could potentially facilitate the formation of structurally defined bio/nano interfaces as well as molecular organization on 2D materials and regulating electronic characteristics of the solid substrate. If achieved, a highly organized peptide array could provide 2D scaffolds for biomolecules towards establishing biological-based 2D nanodevices.

Electronic characteristics of graphene are highly sensitive to adsorbed molecules *via* chemical doping^25^,^26^,^27^. More recently, the doping has also been investigated in single-layer MoS_2_ with its photoluminescence^28^. Extending this strategy, electronic characteristics of 2D single layer materials may also be tuned by adsorbed peptides. Different from simple organic synthetic molecules, peptides have a wide-range of chemistry and molecular folding inherent in their amino acid content and sequence, and play a major role in cell-cell, protein-cell, and protein-DNA interactions^29^. Peptide’s interaction with a single layer material should also be correlated with both its sequence and conformation on the surface.

## Results and Discussion

### Graphite binding peptide and its variants

**Figure 1:**
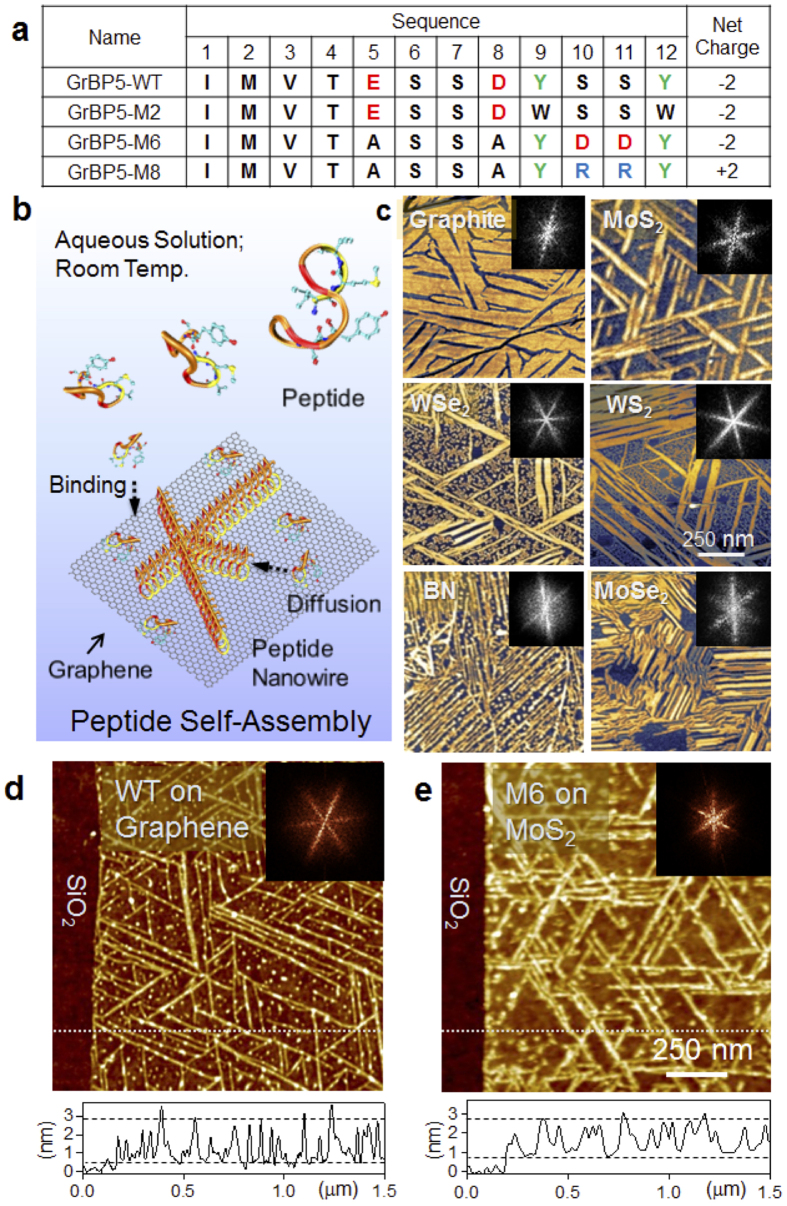
Atomic force microscope (AFM) images of self-assembled peptides. (**a**) Sequences of the Wild Type and variant peptides. (**b**) A schematic showing the peptide self-organization with a series of the surface processes: binding, diffusion and self-organization. (**c**) AFM images of organized peptides on surfaces of bulk graphite, MoS_2_, WSe_2_, WS_2_, MoSe_2_, and hBN. All morphology shows six-fold symmetry. (**d**) AFM of WT on single-layer graphene. The bright lines are self-assembled peptides forming organized nanostructures. (**e**) AFM image of M6 on single-layer MoS_2._ The insets: fast Fourier transform of the AFM image exhibiting six-fold symmetry of the self-assembled peptides.

In our previous work, graphite-binding dodecapeptides (GrBPs) were combinatorially selected using a phage display library^17^. Among these, GrBP5-WT (wild type) peptide forms long-range ordered nanostructures on freshly cleaved graphite (Fig. 1). Peptide adsorption is accompanied by a series of surface phenomena including binding, diffusion, and assembly (Fig. 1b), each of which can be tuned by simple point mutation in the original sequence^17^. In this work, we designed dodecapeptides (Fig. 1a) to form organized structures on 2D materials: graphite, MoS_2_, WSe_2_, WS_2_, MoSe_2_, and hBN. Atomic force microscopy (AFM) images show peptide organized structures with six-fold symmetry (Fig. 1c). A simple procedure of placing an aqueous droplet of peptide solution allows the biomolecules to spontaneously adsorb on the surface and spread to form monolayer (1–2 nm) thick nanostructures. We use graphene and MoS_2_, respectively, as representatives of semimetallic and semiconducting 2D nanomaterials to investigate modulation of their electrical characteristics by peptides. As we demonstrate below, a simple mutation of the sequence induces peptides to assemble in random *vs.* ordered organization with significantly different resultant effects, examined by field effect transistors (FETs) of graphene and MoS_2_.

### Peptide nanowire formation on single-layer graphene and MoS_2_

First, we demonstrate that GrBP5-WT also spontaneously forms ordered structures even on single-layer graphene in aqueous solution at room temperature (Fig. 1d). AFM imaging reveals that GrBP5-WT self-organizes forming “nanowire” architectures on graphene with six-fold symmetry (Fig. 1d) commensurate with the underlying honeycomb carbon lattice. The nanowire longitudinal length ranges up to micrometers, while the average thickness (height of peptide) is 1.1 nm. There are no adsorbed peptides on SiO_2_, indicating they only specifically bind to graphene.

Instead of nanowires on graphene, the WT peptide forms confluent disorganized film on single-layer MoS_2_ (Supplementary Information S3, S4, and Figs S2–S5). To maintain organized structures on MoS_2_, we rationally designed dodecapeptides, GrBP5-M6 and -M8, by site-directed mutagenesis of the WT as the primary sequence (Fig. 1a,d). First, in M6 variant, the motif “YSSY,” previously identified in WT as putative binding domain to graphite *via* π-electrons of phenyl in Tyrosine, was replaced with “YDDY,” containing two negatively charged Aspartates to enhance electronic interactions by bringing the charged moieties closer to the solid surface. Second, to maintain the original net charge of WT, the central Aspartic (D) and Glutamic acid (E) residues were replaced with Alanine (A), a charge-neutral amino acid. Similarly, M8 was designed with “YRRY”, containing positively-charged Arginine (R) within the binding domain.

To test their ability, we incubated the variant peptides on bulk samples. AFM reveals that our strategy with M6 (net -2 charge) worked remarkably well in the formation of nanowires on MoS_2_ and high aspect ratio structures even on the other layered materials: MoSe_2_, WS_2_, WSe_2_, and BN (Fig. 1c). Similar to WT on graphene, M6 also forms nanowires on single-layer MoS_2_ (Fig. 1e). The morphology of the peptides has six-fold symmetry on all surfaces, possibly conforming to the hexagonal 2D lattices. The variant M8 with a positive net charge, however, does not form ordered structure but a confluent disordered films on all the 2D layered materials (Supplementary Information S2, S3, S4 and S6). These observations suggest that even a slight change in the amino acid sequence leads to alterations in peptide-surface and intermolecular interactions, possibly induced by favourable folding conformations on surfaces followed by constructive molecular recognition.

### Modulation of electrical conductivity of graphene by peptides

**Figure 2:**
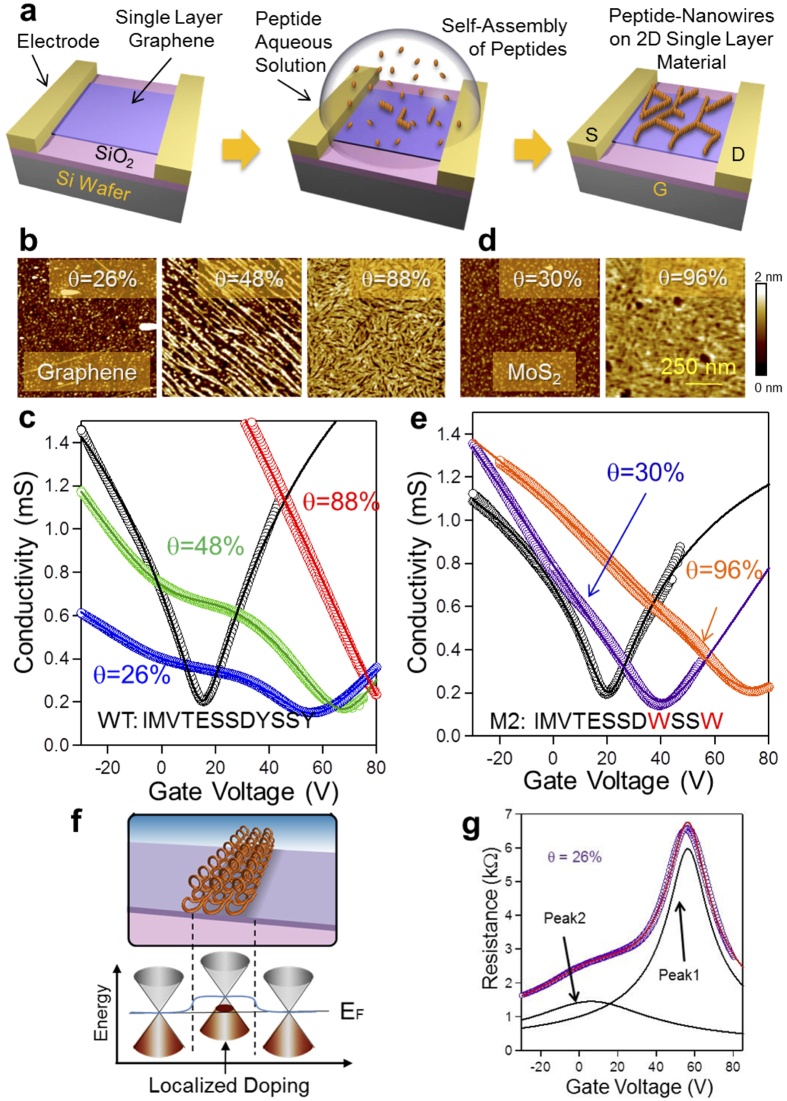
Peptide nanostructures on GFETs and their corresponding conductivity characteristics. (**a**) A schematic of peptide assembly on a GFET. A droplet of peptide aqueous solution is placed on a GFET and then dried by nitrogen blow. (**b**) AFM images of GrBP5 peptides on GFETs with various peptide coverages from 26% to 88%. (**c**) The conductivity *vs.* back-gate voltage for as-prepared GFET (black) and graphene FETs with different peptide coverages corresponding to AFM images in (**a**). (**d**) AFM of GrBP5-M2 on GFETs with coverages of 30% and 96%. (**e**) The conductivity *vs*. back-gate voltage for GFETs with GrBP5-M2. The marks in the plots represent experimental data points and solid lines represent fitted curves. (**f**) A schematic showing spatial doping of graphene by peptide nanowires. (**g**) Resistance *vs*. gate voltage of GFET with 26% peptide coverage shows a good agreement in fitting with two peak components.

We next characterized graphene conductivity of FET devices (GFET) upon peptide assembly (Fig. 2a). While peptide coverage is controlled by incubation time and concentration, exceptional care was taken to ensure reproducible assembly on active GFET surfaces and subsequent conductivity measurements (Methods Summary and Supplementary Information S5–S7). Similar to the surface phenomena observed on graphite^17^, peptide structures on graphene change from islands to nanowires with increasing coverage (Fig. 2b), eventually forming confluent monomolecular film. The gate-voltage response of the GFET conductivity clearly shows modulation by the adsorbed peptides for a given coverage (Fig. 2c). All as-prepared GFETs show a charge neutral point (CNP) at around 20 V, *i.e.*, the voltage with minimum conductivity, similar to those reported for graphene on SiO_2_ 6. Surprisingly, the GFETs with peptide coverage of 26% and 48% display two distinct dips in conductivity, *i.e*., two CNPs. As the coverage increases, the left dip gradually disappears while the right one becomes more dominant. At 88% coverage, the overall dip shifts to more than +80 V. Conductivity does not display hysteresis upon cyclical sweep of the gate voltage, suggesting that peptides are rigidly immobilized and stable even under an electric field. The presence of two dips is unique in graphene with self-organized peptides compared to previous reports on chemical doping of graphene^25^,^26^,^27^. It was shown that a single-dip CNP monotonically shifts without displaying a second dip, as atoms or organic molecules are randomly deposited on graphene. Two minima in the conductivity have been observed in micro-patterned GFETs, where chemical dopants^26^ selectively cover part of graphene while remaining is uncovered. Our observation of two minima suggests that peptides spontaneously form two electrically distinct regions in graphene (Fig. 2f); uncovered region without doping and peptide covered region with hole doping.

The implication of the organized peptides on graphene conductivity was further examined by control experiments with a variant peptide M2 (IMVTESSDWSSW). Here, Tyrosines (Y), with phenyl group in the WT’s binding domain, are replaced by Tryptophans (W) containing indole moiety. Possibly due to strong binding affinity of Tryptophan on graphene, M2 forms disorganized nanostructures, *i.e.*, “random islands”, without the symmetry of the underlying solid lattice (Fig. 2d). Correspondingly, the measurements of M2-GFET conductivity *versus* gate voltage display a single dip, rather than two seen in WT-GFET. Also, the CNP shifts up to 75 V as the coverage gradually increases to 96% (Fig. 2e). The absence of two dips, therefore, may be due to random doping by disorganized M2 structures.

**Figure 3:**
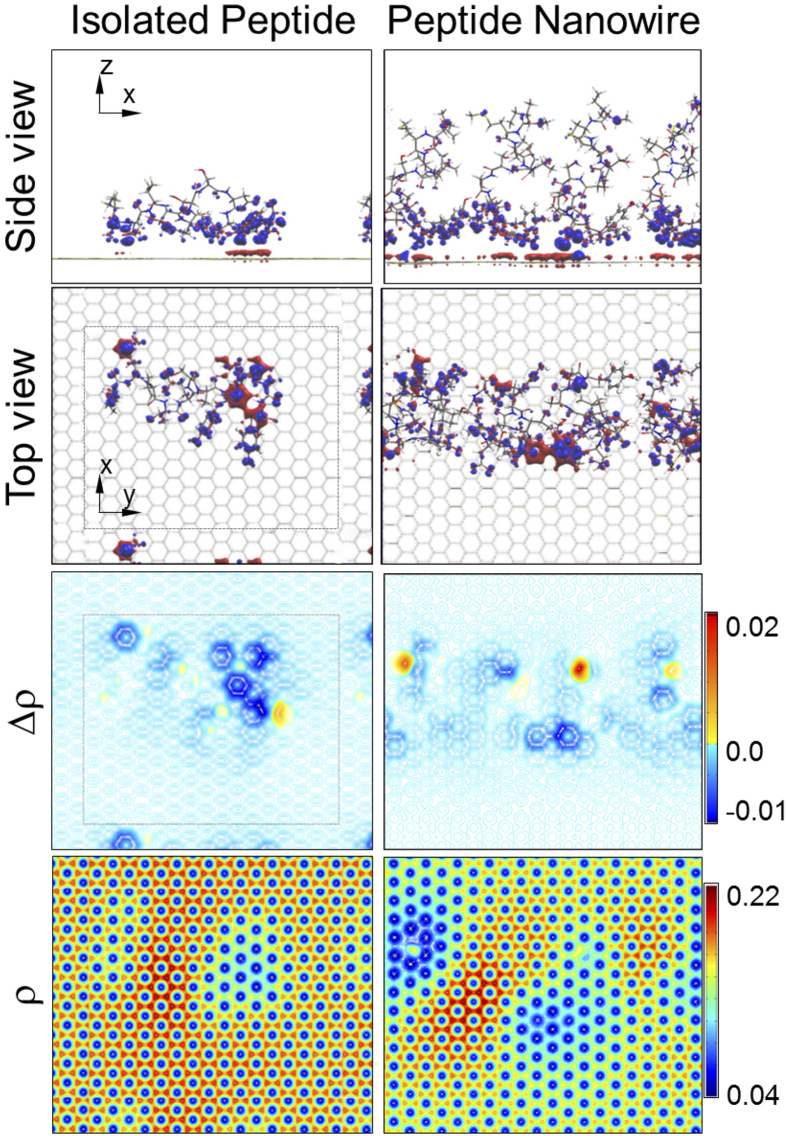
Computational modeling with molecular dynamics and density functional theory for two models. One is an isolated peptide on a graphene and the other is a peptide nanowire consisting of four peptides. Δρ shows the charge transfer from the graphene to peptides while ρ represents total charge in graphene.

To investigate the observed effects further, we quantitatively analyze graphene conductivity by fitting with a well-accepted equation, representing series resistance of two independent regions in a GFET (Fig. 2f,g) (see Methods Summary and Supplementary Information S8). The fitted curves display good agreement with the measured conductivity with two dips suggesting that the peptide-modified graphene has two distinct regions (1 and 2) with independent electrical characteristics. Also, the charge carrier mobility exhibits a clear difference between WT and M2 (Supplementary Information S8). While M2 shows substantial decrease to 1,800 from 4,300 cm^2^/Vs (as-prepared GFETs) and stays constant over the full range of coverage, the mobility modified by WT decreases to 300 cm^2^/Vs at 20% coverage and gradually recovers up to 2800 cm^2^/Vs as coverage increases and peptides assemble into more ordered nanowires. Similar to the reduction in mobility observed by chemical adsorbates on graphene^25^, randomly adsorbed peptides may act as scattering points. The recovery of the mobility revealed here has so far not been reported in a GFET with increased amount of adsorbate molecules. Although the mechanism of change in mobility by impurities is still under discussion^30^,^31^, our observation may be related to the screening effect, where dielectric media such as solvents^32^ and surfactants^33^ on graphene can have a less effect of charge scattering because of these molecules forming soft interfaces through weak molecular interactions, as opposed to covalently bonded adsorbates which cause surface defects, and hence become scattering centers. The ordered peptides used here have densely-packed nano-structures with soft, coherent interfaces and high dielectric constant (Fig. 3). In contrast to the recovery of the mobility by WT peptide, the mobility of the GFETs constructed with the M2 variant displays monotonic decrease with increased peptide adsorption. These results suggest that while molecular recognition of the graphene crystal lattice by the WT peptide leads to the formation of a coherent interface with substrate-templated organized peptides, random organization of the M2 peptide leads to disorganized (amorphous) peptides that act as scatterers degrading the mobility.

### Computational simulations of peptides on graphene

To gain better insight into possible effect of peptide molecular folding and subsequent ordering, we carried out DFT (Density Functional Theory) simulation^34^, which is known to provide charge transfer and local carrier density in graphene (Supplementary Information S12). Two cases were modelled: single peptide and molecular wire consisting of four peptides in a cell (Fig. 3). The calculation shows four peptides “stand up” and form a tightly packed structure while a single, isolated peptide lies down and strongly binds to graphene. The DFT results are consistent with AFM height measurements of isolated or small peptide clusters, (1.1 nm) and ordered peptide nanowires (1.4 nm) (Supplementary Information S10, and Figs S12 and S13). Regarding the charge transfer, the calculations have two key implications. First, both models show “hole doping” into graphene, agreeing well with our experimental observations. Second, the four-peptide model shows a larger total charge transfer than the total of separated four single peptides, suggesting that ordering forms a stronger total dipole moment in a coupled peptide nanostructure, such as a nanowire.

### Electrical and optical properties of MoS_2_ modulated by peptides

**Figure 4:**
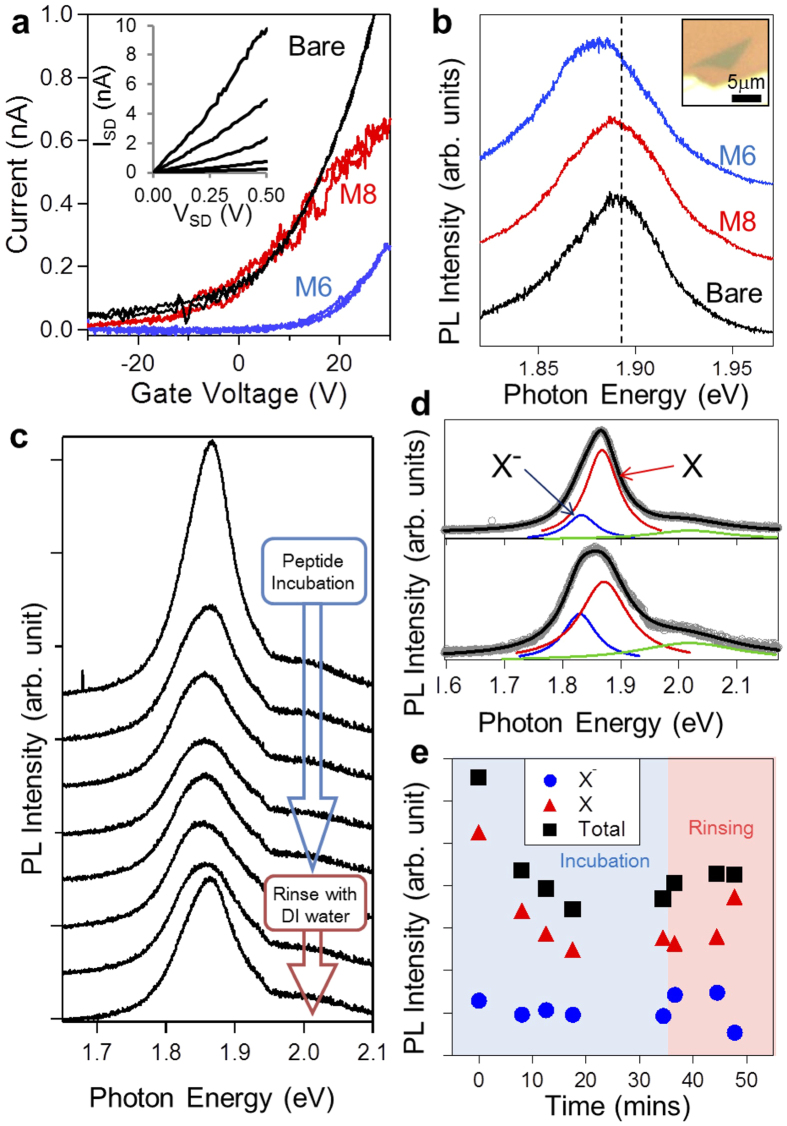
Characterization of single-layer MoS_2_ modified by peptides. (**a**) The source-drain current *vs*. back-gate voltage of single-layer MoS_2_ FETs before (black) and after incubation with variant peptides M6 (blue) and M8 (red). The source-drain voltage was 0.1 V. The inset shows a plot of source-drain current *vs*. source-drain voltage with various back-gate voltages of −20, −10, 0, 10, and 20 V; the observed current increases, corresponding to the gate voltage. (**b**) Photoluminescence spectra of single-layer MoS_2_ before (black) and after incubation with variant peptides M6 (blue) and M8 (red). The inset shows an optical image of single-layer MoS_2_. (**c**) *in-situ* PL spectra during M6 variant incubation and rinsing. (**d**) Peak fitting with three Lorentzian peaks shows contributions of free excitons (X) and trions (X^−^). Top and bottom spectra show before and after peptide incubation. (**e**) Integrated intensity of each peak X (red) and X^−^ (blue) after incubation and rinsing. Black marks show the total intensity of the PL (X + X^−^). The X intensity decreases during peptide incubation, and recovers slightly after rinsing with DI water. For all PL measurements, the excitation wavelength and power were 532 nm and 10 μW, respectively.

In light of the effects of organized *vs.* disorganized peptide nanostructures on graphene conductivity, we next examine the same in single-layer MoS_2_. Mechanically exfoliated single-layer MoS_2_ is used in FET configuration to quantify conductivity modulated by the peptides (Supplementary Information S5). In gate response, as-prepared MoS_2_ FETs behave as an n-type channel, similar to previous reports^20^. Upon incubating with M6 variant peptides, however, MoS_2_-FET displays hole doping as a shift in threshold voltage from −10 V to 15 V (Fig. 4a). The use of M8 variant, however, causes no shift. Both peptides exhibit similarly diminished slopes as compared to bare MoS_2_, indicating decrease in carrier mobility. The photoluminescence (PL) spectra obtained from peptide-functionalized MoS_2_ display a similar tendency as the conductivity measurements. While M6 shows a large peak shift of 9 meV in the PL spectrum, M8 exhibits no shift (Fig. 4b). To examine the origin of peak shift by M6, we monitored *in-situ* PL spectra of MoS_2_ under peptide solution. Our observations demonstrate good agreement with previous studies describing the presence of trions in MoS_2_ 28. After incubating M6, the strong original PL intensity from free excitons is gradually suppressed; however, it recovers after rinsing peptides with DI water (Fig. 4c–e). Peak fitting with Lorentzian confirms good matching with contributions from free excitons and trions, respectively (Fig. 4d). The suppression of the free excitons may indicate that organized peptides facilitate the capability to control carrier density of MoS_2_ even under solution condition (Fig. 4e).

## Conclusions

We show here that peptides can form organized biomolecular nanostructures on single-layer graphene and MoS_2_, possibly a result of lattice matching enabled by refolding of the relevant peptide upon molecularly recognizing the 2D surface lattice, thereby creating a coherent bio/nano interface. Avoiding defect formation on the surface and, hence, no charge scattering, the weak peptide-solid interaction facilitates uniform doping of the 2D materials maintaining their intrinsic electrical (graphene) or optical (MoS_2_) characteristics. The work opens up potential utility of 2D nanomaterials in biology and medical implementations^35^ enabled by the controlled interface modification by the engineered peptides. Arrays of peptides, for example, could provide organized scaffolds for functional biomolecules, enabling nanoscale bioelectronics interface. The opportunities provided by peptides could also extend beyond simple doping. Designed peptides can be incorporated with metal ions or nanoparticles with specific physical characteristics, thereby fine-tuning 2D device performance^14^. Controlled immobilization of heterofunctional chimeric peptides incorporating (chemical or biological) probes could be used for enhancing functionality of 2D materials towards chemical and biological sensors. Finally, the coherent bio/nano interfaces are also analogous to another famous materials science problem of several decades ago, namely coherent interfaces in compound semiconductors developed during the 80 s that enabled today’s electronics^36^. It is conceivable that by more detailed understanding of the intricacies of the coherent bio/nano interface structures, the effects of electrochemical conditions, molecular recognition characteristics of different peptide sequences, and physical changes in the 2D material across the interface, we can make major strides in the *in vivo* integration of the worlds of biology and solid-state devices of the future.

## Methods

### Conductivity Measurement

Special care was taken for reliable characterization of graphene conductivity induced by the ordered peptides. First, GFETs are fabricated by direct soldering of indium contacts to an exfoliated graphene on a SiO_2_/Si wafer. We employed this resist-free process, instead of a conventional lithography, because of the difficulty to completely remove residues from the resists that disrupt peptide self-assembly into ordered structures. Second, we directly place a droplet of peptide aqueous solution on GFETs to prevent contamination. After the incubation, samples are quickly dried by nitrogen blow. In the solution process, water molecules are known to significantly change the conductivity of the graphene on SiO_2_ in the presence of oxygen. To prevent the water effect, we utilize deoxygenated DI water prepared by bubbling with nitrogen or argon gases. All the conductivity measurements, therefore, have been performed in argon atmosphere to eliminate the water effect. In the conductivity measurement, gate-voltage was cyclically swept over a range from 0 V to maximum, from maximum to minimum, and back to 0 V.

We use a modification of the original equation (Supplementary Information S8) for the observation, as below.


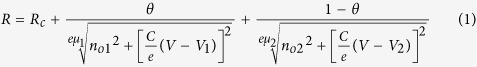


where, *R* is total resistance of graphene, *R*_*c*_ is contact resistance, *e* is electron charge, μ_1,2_ is charge mobility, *n*_*o1*_*, n*_*o2*_ are residual density of electron-hole puddles on SiO_2_ that screen the electric potential from the bottom gate, *C* is capacitance of the GFET, *V* is gate voltage, θ is the coverage of the peptides on graphene, and *V*_*1,2*_ is CNP.

## Additional Information

**How to cite this article**: Hayamizu, Y. *et al*. Bioelectronic interfaces by spontaneously organized peptides on 2D atomic single layer materials. *Sci. Rep.* **6**, 33778; doi: 10.1038/srep33778 (2016).

## Supplementary Material

Supplementary Information
