# Supplementary material for: Bioelectronic interfaces by spontaneously organized peptides on 2D atomic single layer materials
Source: Sci Rep. 2016 Sep 22;6:33778. doi: 10.1038/srep33778 (PMC5031961; doi:10.1038/srep33778)
Supplement: Supplementary Information [file srep33778-s1.doc]

**Bioelectronic interfaces by spontaneously organized peptides on 2D atomic single layer materials**

***Yuhei Hayamizu,^1,2,3^  Christopher R. So^1^, Sefa Dag,^1^ Tamon S. Page,^1^ David Starkebaum,^1^ and Mehmet Sarikaya^1*^***

^1^Genetically Engineered Materials Science and Engineering Center, MSE, University of Washington, Seattle, WA 98195, USA;

^2^Department of Organic and Polymeric Materials, Tokyo Institute of Technology, Tokyo 152-8550, Japan;

^3^PRESTO, Japan Science and Technology Agency (JST), 4-1-8 Honcho, Kawaguchi, Saitama 332-0012, Japan.

***Contact author: sarikaya@u.washington.edu***

The comprehensive supplementary information given here gives both further support for the results, discussions, and interpretations made in the main text of the manuscript as well as provides experimental and procedural details.

**S1. Peptide synthesis**

Peptide synthesis was carried out on a preloaded support resin using HBTU activation chemistry. 20% piperidine in DMF was used for Fmoc deprotection and the reaction efficiency was monitored by UV absorbance at 301 nm. The peptides were then cleaved from the resin and side chains deprotected *via* a cleavage cocktail under N_2_ atmosphere for 2-3 hours. The cleavage cocktail contained either TFA/thioanisole/H_2_O/phenol/ethanedithiol (87.5:5:5:2.5) or TFA/triisopropylsilane/H_2_O/EDT (94:1:2.5:2.5), depending on the peptide. The resin was removed by filtration, and each of the peptides was precipitated with cold ether to yield crude peptide product that was lyophilized (Virtis Benchtop K, SP Industries, Inc., Warminster, PA). Peptides were reconstituted using various ratios of DI water and acetonitrile. Purification by reverse-phase HPLC for peptides employed, first, an isocratic (0% B for 2 min.) and, then, a linear gradient of 1%/ min for analytical and 0.5%/min for semiprep scales at 1 and 10 mL/min flow rates, respectively. Retention times spanned 30-50 minutes depending on the peptide in semi-preparative HPLC. Analytical peaks were isolated by autothreshold collection (Waters Deltaprep 600, analytical mode) and peptides were verified by MALDI-TOF mass spectrometry with reflectron (RETOF-MS) on an Autoflex II (Bruker Daltonics, Billerica, MA) mass spectrometer in positive-ion mode. The observed M/Z fractions were subsequently collected manually from a scaled semi-preparative separation (Waters Deltaprep 600, semiprep. mode).


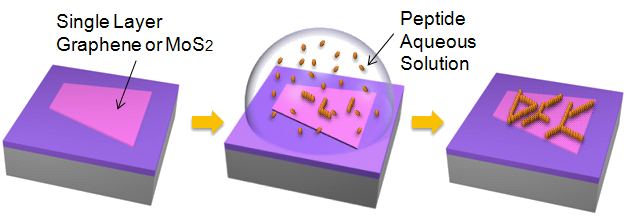


**Figure S1.** Sample preparation of peptide assembly on graphene or single layer MoS_2_.

**S2. Sample preparation for atomic force microscope studies**

Figure S1 shows schematics of the sample preparation for graphene with peptides. Graphene was mechanically exfoliated from natural graphite flakes (3763, Asbury Carbons) on a Si wafer with 300 nm SiO_2_. The number of graphene layers was confirmed by Raman spectroscopy (Supplementary Information S10 and Fig. S15) and atomic force microscopy (AFM). Single-layer MoS_2_ was also mechanically exfoliated from bulk MoS_2_ (Moly Disulfide, SPI Supplies). The MoS_2_ samples being monolayer were confirmed by AFM and photoluminescence (PL) measurements. After exfoliation, samples were incubated with peptides in DI water. The samples for Fig. 1c were incubated by each peptide solution under the condition of 1 μM for 1 hour: WT is for graphite; M6 is for MoS_2_, MoSe_2_, WS_2_, WSe_2_, and BN. The sample for Fig. 1d was incubated under the condition of 500 nM WT peptides for 20 minutes. The sample for Fig. 1e was prepared under the condition of 1 μM M6 peptide for 1 hour. After the incubation, samples were gently dried under nitrogen flow.

**S3. Acquisition of AFM images and analysis of peptides on graphene and MoS_2_**

To quantitatively compare the thickness of adsorbed peptide structures on graphene and MoS_2_, we further analyzed the height histograms of AFM images displayed in Fig. 1. Figure S2 and S3 show AFM images and their corresponding height histograms for the three peptides used herein (WT, M6 and M8) on graphene and MoS_2_, respectively. These histograms were obtained using the AFM data analysis software SPIP. Each histogram was fitted with a Gaussian peak to derive the average thickness of peptide structures. In this analysis, most images are best fit with three Gaussian peaks, except for WT on a graphene. In addition to the bare surface peak, the two other individual peaks in the histogram may indicate that AFM images contain distributions of structures on surfaces that correlate to the two distinct morphological phases: disordered (random adsorption and distribution of peptide, and ordered, patterned assembly of peptides on the surface. In the case of the WT peptides on graphene, the AFM image does not display disordered peptides. The fitting results clearly show that peptides have a similar thickness of about 1 nm on both graphene and MoS_2_, with the exception of M8 on MoS_2_, with a thickness of 1.7 nm. This observation implies that the distinct effects of M6 and M8 on the PL and conductivity of MoS_2_ in Fig. 4 originates from a conformational difference between M6 and M8 on MoS_2_.


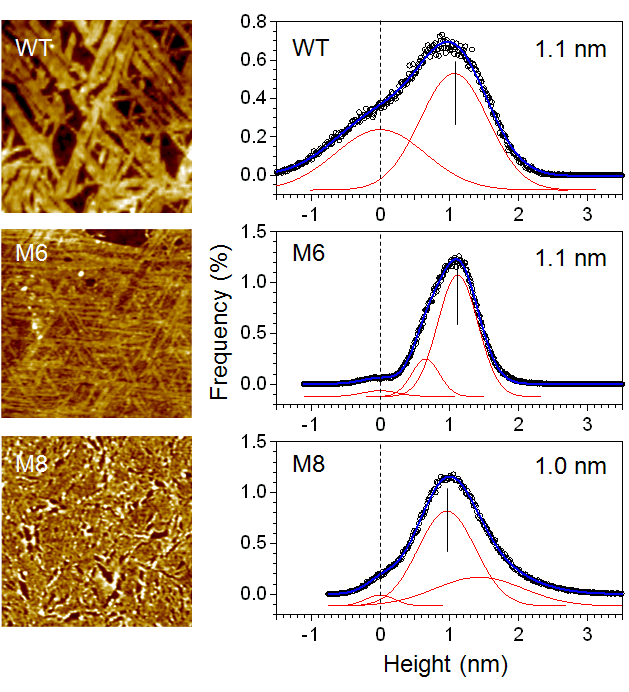


**Figure S2.** AFM images of peptides on single-layer graphene (left side) and their height histogram (right side). The size of each AFM image is 1 by 1 μm. Dashed lines in the plots indicate the measured height of the graphene surface on average. In the histogram, WT has two peaks of bare graphene surface and peptide nanowires. M6 shows three peaks from bare graphene surface and two phases of the peptides (thick and thin phases). The thick phase of peptides shows ordered structures, and is dominant in the histogram. M8 also shows two phases of peptides without ordered structures, and thin phase is dominant. The thick phase is formed at the edge of peptide film.


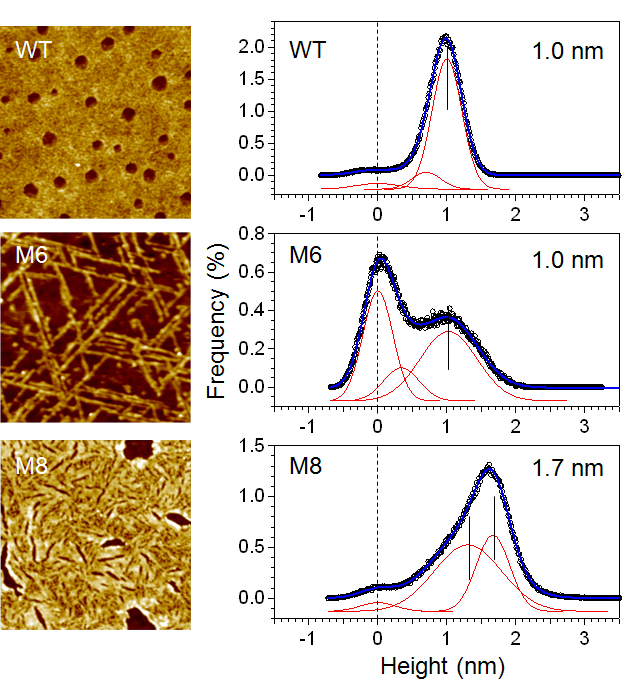


**Figure S3.** AFM images of peptides on single-layer MoS_2_ (left side) and their height histogram (right side). The size of each AFM image is 1 μm by 1 μm. Dashed lines in the plots indicate the measured height of the MoS_2_ surface on average. WT formed a confluent film on MoS_2_ with some pores. M6 forms nanowires and some isolated peptides on the surface. The isolated peptides are thinner than nanowires, and nanowires are dominant in the histogram. M8 shows two phases of peptides. Similar to the M8 on graphene, thicker phase is formed at the edge of peptides film. Both phases of M8 peptides are thicker than other peptides, WT and M6.


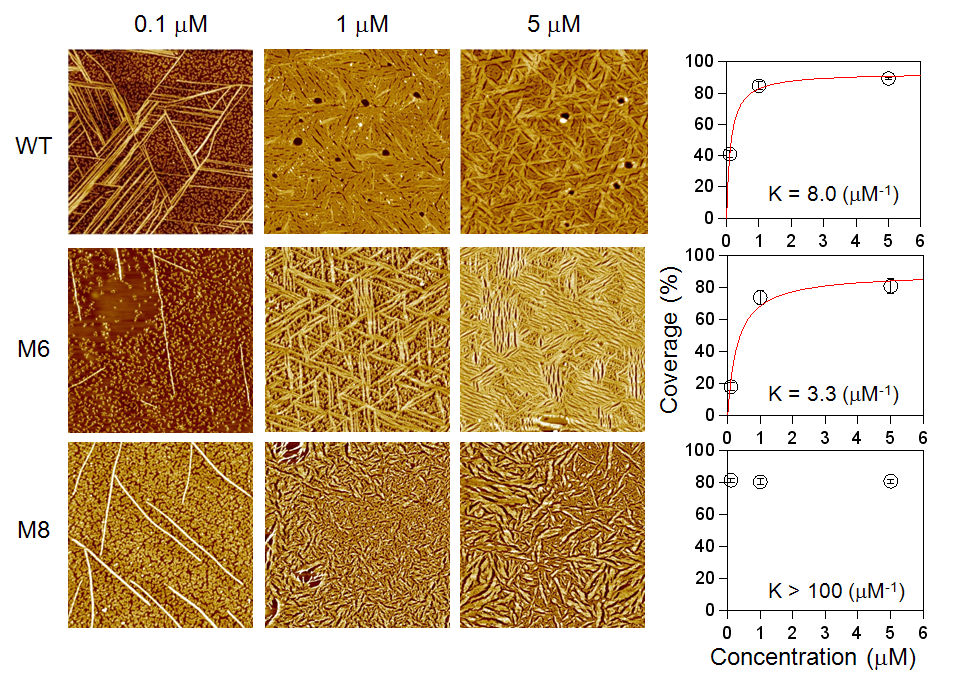


**Figure S4.** AFM images of peptides on surfaces of graphite and the plots of coverage of peptides vs. incubation concentration of peptides. The size of each AFM image is 2 μm by 2 μm. The high coverage of M8 for all concentrations does not allow us to estimate a quantitative binding affinity constant. However, the K for M8 cannot be less than 100 μM^-1^ from the observed coverage within the concentration range.

**S4. Characterization of GrBP5 and its variants, M6 and M8, on bulk graphite and MoS_2_**


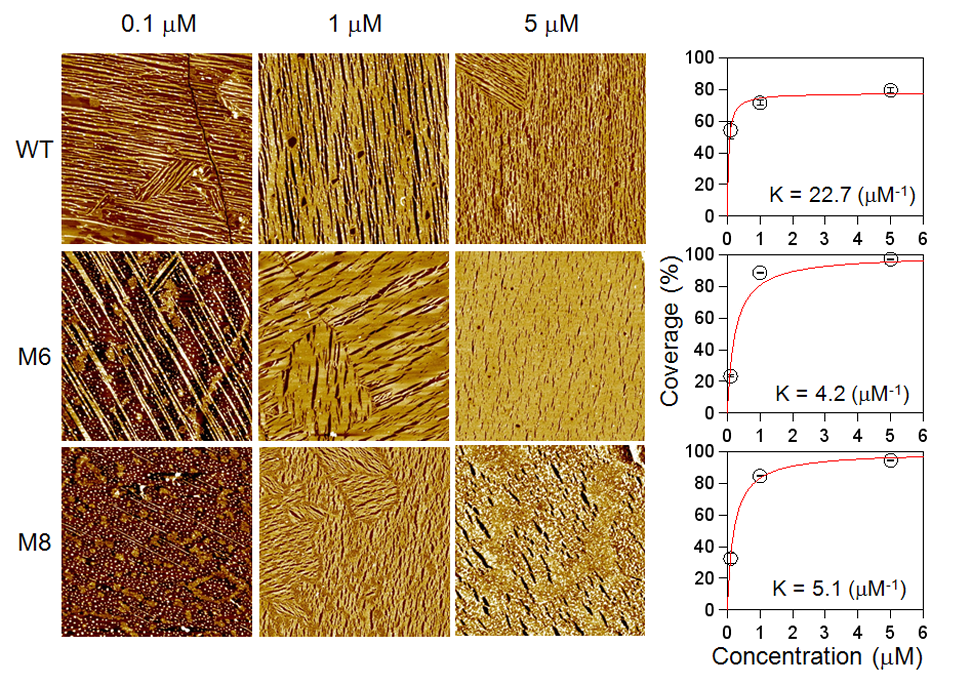


**Figure S5.** AFM images of peptides on bulk MoS_2_ surface and the plots of coverage vs. incubation concentration of peptides. The size of each AFM image is 2 μm by 2 μm.

To roughly estimate binding affinities of each peptide (WT, M6 and M8) to surfaces, we characterized them on bulk graphite and MoS_2_ by AFM. Flakes of graphite and MoS_2_ on Si wafers with 300 nm-thick SiO_2_ were incubated with aqueous peptide solution for 3 hours. The concentrations of peptides were 0.1, 1, and 5 µM. After the incubation, the samples were gently dried under nitrogen gas. Figure S4 and S5 show AFM images of peptides, WT, M6 and M8, on graphite and MoS_2_ at each concentration, respectively. Interestingly, the results show each kind of peptides capable of forming long-range ordered nanowires at 0.1 µM concentration on both graphite and MoS_2_, except for M8 on graphite, which instead forms fiber-like structures with a somewhat loose organization on the surface. At higher concentrations, upon adsorption to the surface, all peptides display domains with aligned peptide nanostructures, except for M8 on graphite which form randomly distributed domains. The distinctive behavior of M8 on graphite was also observed in its binding affinity. The binding affinities of peptides to graphite and MoS_2_ were estimated from a relationship between the observed surface coverage of peptides by AFM and the peptide concentrations used for incubation. A simple adsorption relationship, Langmuir adsorption isotherm, was used which allows provides a fairly reliable estimate of the binding affinity, as follows:


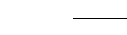


where, θ is surface coverage of peptides, θ_max_ is maximal coverage of peptides, C is concentration of peptide solution, and K is binding affinity constant. The binding affinities obtained by fitting the adsorption equation with experimental data (Fig. S6) show that M8 has an exceptionally large binding affinity to graphite. On the other hand, other peptides, WT and M6, have relatively weak binding affinities. In fact, aligned peptide nanowires on graphite were already observed in previous reports, which characterized surface morphology of peptides on graphite at various conditions^7,8,17^. These reports suggest that binding, diffusion and intermolecular interactions of peptides play important roles during their self-assembly process into long-range ordered structures. It also suggests that strong interactions between peptides and the surface results in suppressed surface-diffusion rates and therefore inhibits peptide self-assembly into ordered structures. In light of such findings, our observations indicate that M8 may have stronger interactions with graphite surfaces, suppressing surface diffusion. WT and M6, on the other hand, may have optimal interactions with the surface, readily forming self-assembled structures. Also notable is the contrast in peptide binding specificity to particular materials that arise from modified primary amino acid sequences. While M6 has weak but comparable binding affinities to both graphite and MoS_2_, WT and M8 show opposing trends in binding affinity. While WT binds strongly to MoS_2_, M8 binds weakly. In the case of graphite, however, this tendency is reversed. The opposing material selectivity of the peptides may be due to the difference in electric potential of graphite and MoS_2_ at the interface between the solid surface and peptide solution. WT and M8, with their opposing mean charge, might therefore show contrasting tendencies in their material selectivity. At the same time, they may have a higher sensitivity to the electric potential of surfaces over M6. Although these hypotheses need to be investigated further, the significant effect on peptide binding affinity after simple modification of the amino acid sequence sheds light on the high level capability of molecular tuning of the peptides’ functions and self-organization onto materials of different chracatristics.


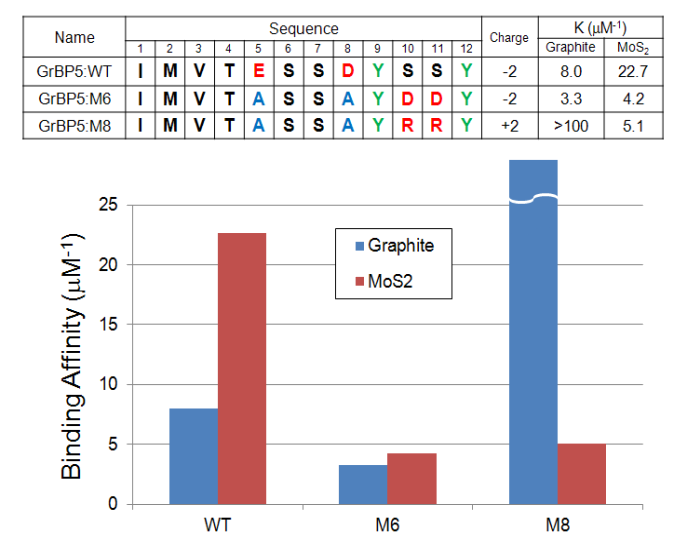


**Figure S6.** Binding affinity of the peptides WT and, M6 and M8 to graphite and MoS_2_, respectively.

**S5. Fabrication of field effect transistors (FET)**


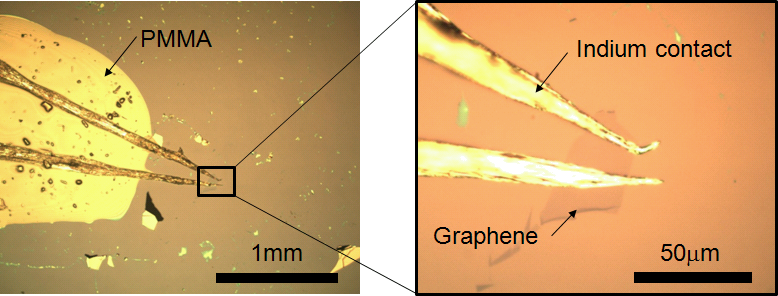


**Figure S7.** Optical microscope images of a typical GFET used for conductivity measurements. Indium electrodes were partially covered by PMMA to prevent undesired electrochemical interactions with water during the peptide incubation.

Graphene was exfoliated from natural graphite flakes (3763, Asbury Carbons) by the conventional mechanical exfoliation method^6^ and supported on a doped Si wafer containing a 300 nm-think native oxide. That the graphene samples are single layers were identified by Raman spectroscopy and AFM. In this study, the cleanness of graphene surfaces was vital in order to ensure (and observe) ordering of the peptides. In conventional lithographic fabrication of graphene FETs (GFET), the residues and artefacts from resist and other preparation material often remain on graphene surfaces even after rigorous cleaning processes. Such uncontrolled contamination was found to cause deleterious doping effects in graphene as well as disrupting the self-organization of peptides on the surface (possibly by disrupting molecular interactions). To avoid such contamination, we utilized micro-soldering of Indium^37^. After mechanical exfoliation of graphene, we immediately formed indium electrodes on graphene. The Indium electrodes were also manually covered by PMMA (495, MicroChem Corp.) to prevent their possible electrochemical effects onto graphene and self-organization of peptides during incubation (Fig. S7). The peptides were immobilized by placing about 20 μL droplets of peptide aqueous solution at various concentrations onto a GFET chip. Samples were kept under humidity control (>80%) chamber to prevent drying during their incubation. The GFETs prepared were then rinsed with DI water promptly and dried with nitrogen flow. The details of the incubation conditions of the images from the fabricated GFETs in Fig. 2b are as follows; Sample 1: θ=26%, 50 nM for 20 minutes; Sample 2: θ=48%, 200 nM for 90 minutes; Sample 3: θ=88%, 500 nM for 20 minutes.

In the case of MoS_2_, due to the difficulty in mechanically exfoliating large single-layer flakes, we employed electron beam lithography to fabricate electrodes instead of the micro-soldering approach described above and then assembled the peptides. For lithography, we used PMMA (495, MicroChem Corp.) as a resist, and exposed it using a scanning electron microscope (SEM7000, JEOL). After development in MIBK:IPA 1:3 solution (developer, Microchem) for 3 min, we sputtered Ti/Pt (5 nm/25 nm) as electrodes, and lifted off by boiling acetone and isopropyl alcohol. All FET devices are cleaned by following three processes; (1) Rinsing with boiling acetone for 30 minutes; (2) Cleaning with UV/ozone (UV/Ozone ProCleaner, BioForce Nanosciences Inc.) for 15 seconds; (3) Current induced annealing under argon gas atmosphere. Following these procedures, we confirmed that conductivity was regenerated in each of the three FET devices by using this cleaning process after the incubation with peptides. In this study, we utilized the same FET platform to directly compare the modification of MoS_2_ conductivity by M6 and M8 peptides. All peptides were incubated with the MoS_2_ FET under a condition of 1 μM for 1 hour. As usual, after incubation, peptide solutions were gently dried by nitrogen.


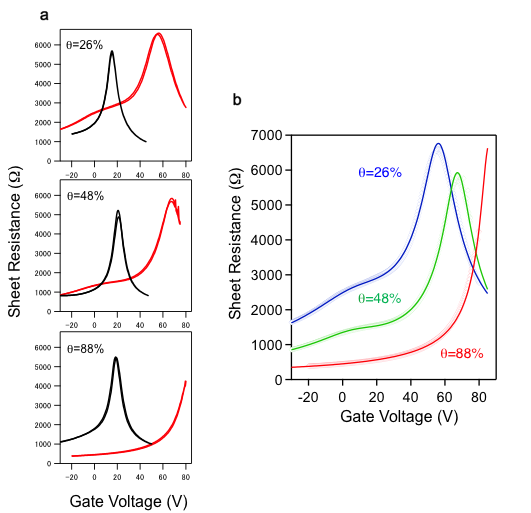


**Figure S8.** Sheet resistance *vs*. gate voltage of graphene FETs shown in Fig. 2: *θ* is coverage of peptides corresponding to the each curve in Fig. 2c. Fig S8a shows raw data of the resistance of GFETs. Black curves show resistance of each GFET before incubation and the red curves show resistance after incubation with the peptides. Fig. S8b shows observed resistance with fitting results.

**S6. Electrical characterization of FETs**

Electric measurements were carried out on two-probe measurements with a semiconductor characterization system (4200-SCS, Keithley). The conductivity of GFETs, Shown, in Fig. 2, was normalized using the dimensions of each graphene sample. The typical size of the graphene sheet between two electric contacts is 40 μm in width and 20 μm in length (Fig. S7). All electrical measurement was performed under Argon atmosphere to eliminate instability caused by moisture in the atmosphere. In gate response measurements, the typical source-drain voltage was 5 mV. In our study, the contact resistance of Indium electrodes was small compared with the resistance of graphene because of the large contact area, typically 40 μm by 10 μm (Fig S7). Typically, resistance of GFET is in the order of kilo-ohms, while contact resistance is sub-ohms. The plots of sheet resistance *vs*. gate voltages also show small contribution of contact resistance (Fig. S8). During conductivity measurements, gate voltages were cyclically swept from 0V to maximal or minimal voltage, and then, back to 0V again.

The conductivity measurement for MoS_2_ FETs was also performed under argon gas. Source-drain voltage was 100mV for gate response measurements.


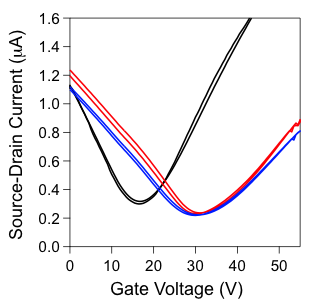


**Figure S9.** Gate responses of GFETs after incubations with degassed DI water: black (as- prepared graphene), red (degassed DI water for 10mins), and blue (degassed DI water for 20mins).

**S7. Prevention of doping by water**

As-prepared GFETs show a charge neutral point (CNP) at around 20V on average. The CNP varies from sample to sample, ranging from 15V to 25V. As observed in previous works^38^, water causes p-type doping for graphene suspended on SiO_2_ in the presence of oxygen. We observed the doping of graphenes in electrical measurements in air and in samples prepared by as-prepared DI water. To prevent the water effect, we used degassed DI water (bubbled with Ar gas for 30 minutes) for all the samples in this work. Figure S9 shows that GFETs incubated in degassed DI water display no significant CNP shift even after 20 mins. of incubation in water. This result indicates that oxygen dissolved in water causes doping of GFET with water. Even after incubation with peptides, GFETs exhibited no hysteresis in cyclic gate voltage measurements. The small shift of the CNP indicates the of doping of graphene. This effect might be caused during transferring the samples from the incubation chamber to the chamber for electrical measurements. The slight shift of the CNP is appears to be significantly smaller than the shifts caused by the peptides.

**S8. Results of the data fitting to the resistivity of graphene FETs**

For curve fitting of our observations, we use the well-known equation as below;


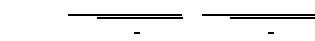
 ,

where, *R* is total resistance of graphene, *R*_c_ is contact resistance, *e* is electron charge, *μ*_1,2_ is charge mobility, *n*_o1_ *and n*_o2_ are residual density of electron-hole puddles on SiO_2_ that screen the electric potential from the bottom gate, *C* is capacitance of the GFET (
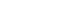
;
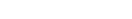
), *V* is gate voltage, *θ* is the coverage of the peptides on graphene, and *V*_1,2_ is the CNP.

Here, the equation represents a series resistance of two independent regions (1 and 2) in a GFET, which can reproduce the two peaks that appear in the resistivity data (Fig. S8). The fitted curves confirm a good agreement with the measured resistivity, suggesting that the peptide modified graphene has two distinct regions, each with independent electrical characteristics, the regions with adsorbed peptides and pristine regions. The question is why the two dips/peaks appear in the conductivity/resistance. To answer this question, we analyze the obtained best-fit parameters of the mobility *μ*_1,2_*,* residual carrier density *n*_o1_*, n*_o2_, and charge carrier density *n*_1,2_, which is proportional to the *V*_1,2_ (Fig. S10). The charge carrier density of *n*_1_ (red dots) extracted from the peak1 increases as coverage increases (Fig. S10a). In contrast, *n*_2_ (green dots) are scattered, but stay constant on average. The observed *n*_1,2_ indicates that the peak1 can be attributed to the resistance component that arises from the peptide-coated regions in graphene, and peak2 can be related to the uncovered regions. When comparing the effects of GrBP5-WT and M2, one notices that both display monotonic increase of hole density as the peptide coverage increases. The increase in both WT and M2 supports our conclusion that these peptides, in general, induce holes into graphene. Also noticed is that WT has slightly higher carrier density than that of M2. This effect might be caused by relatively strong electrical interactions of WT with graphene. In contrast to the carrier density, the mobility exhibits a significant difference between WT and M2 (Fig. S10b). While M2 shows a decrease to 1,800 from 4,300 cm^2^/Vs (as-prepared GFETs) and stays constant over the whole coverage regime, the mobility *μ*_1_* *modified by WT decreases to 300 cm^2^/Vs at the low coverage of 20% and gradually recovers up to 2800 cm^2^/Vs as the coverage increases. The residual carrier densities for each peptide also show a similar tendency. The Peak1 and M2 display relatively constant values over all peptide coverage; however, peak2 appears to have a scattered distribution. This behavior may suggest that peak2 originates not only from the uncovered region of graphene but also from the region with randomly absorbed peptides (white dots in the AFM images).

**Figure S10.** Charge carrier density, mobility, and residual carrier density obtained from fitting with the observations: Black dots represent data points of as-prepared GFETs. Red and green dots are obtained from GFETs with GrBP5:-WT (Fig. 2c). Red and green dots respectively represent the peak1 and peak2 in the plot of resistivity *vs*. gate voltage (Fig. 2f). Blue dots represent data points from GFETs with the peptide variant, M2 (Fig. 2e).

**S9. Conductivity measurements in vacuum**

We characterized the gate response of GFET with peptides in vacuum (Fig. S11). The as-prepared GFET was first measured in our hand-made vacuum chamber with a turbo molecular pump (black curve). Next, the GFET was incubated with 0.5 μM GrBP5 solution for one hour, then dried by nitrogen gas blow. After drying, the sample was placed in the vacuum chamber again. After waiting for three hours, the conductivity was measured (red curve). After overnight pumping, the conductivity was measured again (blue cure).

The charge neutral point of the GFET shows a positive shift after peptide incubation. After further vacuum pumping, the charge neutral point shows slight negative shift from the red curve. The positive shift of the charge neutral point indicates that peptides induce the hole doping in graphene even in vacuum. The slight negative shift after long vacuum pumping implies that decrease of the level of hole doping due to the desorption of adsorbed water molecules. As well as the effect of adsorbed water molecules on the electrical conductivity of graphene, these vacuum measurements support our experimental results showing hole doping by adsorbed peptides.

**Figure S11.** Gate response of a GFET in vacuum.

**Figure S12.** Height analysis of the self-assembled structures of WT peptide on graphene *vs*. on their lateral size. (a) A raw AFM image of peptide nanostructures (nanowires) on a graphene. (b) Automatic recognition of individual peptide nanostructures by SPIP labeled with different colors. (c) A plot of height *vs.* structure length of peptide nanowires. Red and blue dots show maximal and average height of individual nanostructures, respectively.

**S10. Height analysis of peptides on graphene and graphene FETs**

Various peptide nanostructures observed in this study exhibited a height distribution dependent on their lateral size. We analyzed AFM images with peptide nanostructures on single-layer graphene using a particle analysis module in SPIP (Image Metrology A/S) as shown in Fig. S12. The AFM images (Fig. S12a and b) display peptide nanowires to have a linear shape with a lateral length of greater than a micrometer, as well as particle-like islands with a lateral diameter of less than 10 nm. As shown in the plot (Fig. S12c), an increase of lateral size from nanoislands to nanowires correlates monotonically with the height of the nanostructures. This tendency of the peptide morphology was also observed on actual GFET devices (Fig. S13).

**Figure S13.** Height analysis of WT peptide self-assembled structures on GFETs. (a) A plot of maximum height of peptide nanostructures *vs*. the length of the morphological feature on the graphene substrate surface. Here, we extracted data points from all GFETs that were measured in Fig. 2. (b) Average height *vs*. structure length in the same manner of (a).

**Figure S14.** Height analysis of the self-assembled peptide morphologies on GFETs. (a) A plot of height histograms of the WT peptide assembled on graphene. Here, the data points were extracted from the GFET with 48% coverage in Fig. 2b. (b) Average height *vs.* peptide coverage for WT (red) and M2 variant (blue) peptides. WT displays a maximum at around 50%, while M2 increases monotonically with the coverage. These results may be interpreted as follows: The height gradually increases for the WT as the number of peptides increases due to crowding, then has a conformational change, *i.e.*, phase transition (from disordered to ordered) at around 50% coverage and form ordered structures at higher coverage values. In contrast, M2 does not have the conformational change (and, hence, no ordering), but only crowding as the peptide coverage on the surface increases.

**Figure S15.** Phase transition of WT peptides with increasing peptide coverage on the GFETs. (a) and (b) display striking transition from island to nanowire structures of peptides at 40-50% coverage. This result is consistent with the height transition observed in Fig. S14. The coverage characteristics of the peptides in island and nanowire morphologies are characterized by SPIP software (see text for details).

**Figure S16.** Raman spectra of pristine graphene (black) and graphene with adsorbed peptides (red). (a) Typical Raman spectrum of a graphene sheet on Si wafer with 300nm oxide layer on top. The inset shows an optical image of a single-layer graphene recorded by using a green filter. (b) Raman G-band peaks of graphene before (black) and after (red) the incubation with the peptide GrBP5-WT. (c) Same spectra as in (b) showing 2D-band peaks. Solid lines are fitting curves with Lorentzian function.

**S11. Raman measurements for graphene and peptide modified graphene**

Mechanically exfoliated single-layer graphene on Si wafers were verified by Raman spectroscopy based on the recent developments of studying the phenomenon of doping of graphene by organic molecules by this technique^39^. We characterized Raman spectra of graphene before and after incubation with the GrBP5-WT peptide (Fig. S16). Raman spectroscopy was performed using a Renishaw Raman microscope with the 514 nm excitation laser. The excitation power was 100 μW. Fig. S16a displays a typical Raman spectrum of one of our graphene sample^40^. The Si peak at 520 cm^-1^ was used as a reference for wavenumber calibration. Ten graphene samples were used for Raman characterization of the doping effect by the peptides. The well characterized graphene samples were incubated with 1 μM peptide solutions of GrBP5-WT for 1 hour. On average, GrBP5-WT shows 5 cm^-1^ shift in the G-band and 1 cm^-1^ shift in the 2D-band after incubation resulting in surface adsorption.


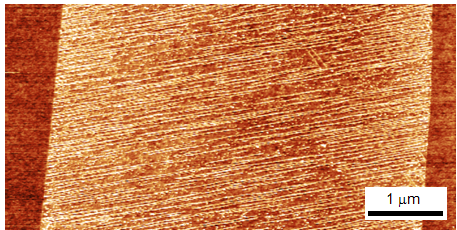


**Figure S17.** An AFM image of self-assembled GrBP5-WT peptide on a single-layer graphene illustrates highly parallel nanowires extending from one edge of the graphene to the other. The incubation condition for the peptides was 100 nM for a period of 5 minutes. These arrays of peptide nanowires, which are potentially useful for a variety of implementations such as biosensors, can be formed by controlling the width of the graphene sheet and the incubation conditions (e.g., peptide concentaration and time).

**S12. Computational methods: Electronic interaction of peptides with graphene**

First-principles total energy and electronic structure calculations have been performed to develop a better insight into the electronic effects of a single peptide molecular adsorption *versus* peptide molecular nanowire on the graphene substrate in the FET devices^41^. It should be noted that when a single peptide is adsorbed onto the surface, it may have a random orientation with respect to the underlying graphene lattice while a nanowire is always aligned with a crystallographic directions of graphene, as experimentally observed. Here, for simplicity, the isolated peptide is also taken to be aligned with the graphene lattice in the same as that of a nanowire as the purpose in computational study has been only to understand the effect of a single versus multimolecule peptide on the interface electronics. Accordingly, first, we examined a possible molecular orientation of a single peptide on the substrate, determined its molecular dipole moment, and its possible effect on the change in the work function of graphene. In the second stage, we compared the value of single peptide to that of peptides in the ordered state, *e.g*., nanowire architecture. To determine molecular folding of the peptides, we initially performed classical molecular dynamics simulations (CMD) with the well-tested CLASS II force field parameters^42,43^. The relaxation calculations were performed with LAMMPS code on the CRAY XE6 machine at NERSC^44^. For this, the molecular structure was first relaxed in an aqueous environment. To determine the total dipole moment of the nanowire, and its effect on graphene, it is necessary to know the molecular folding of the peptides that make up the nanowire so that the periodicity of molecules on graphene surface can be predicted and its consequent effect can be examined. For an estimate of the geometry of the folded peptides, we used the results of the experimental height analysis of the peptides that are organized on graphene; these values enabled us to predict a possible folded structure of peptides within a nanowire architecture. According to the height analysis of the bound peptides, the tail of each peptide (i.e., IMVT..) should be away from the surface while the anchoring domain, YSSY, should be in contact with the surface. For simplicity, nanowire is assumed to be aligned along the zigzag direction of graphene, corresponding to the experimental results that nanowires form a six-fold patterned structure on graphene lattice which also has simple 6-fold crystallographic symmetry. The calculations, carried out accordingly, provided the number of molecules on a graphene supercell structure. For calculations, 40 Å vacuum space has been used in perpendicular, {z}, direction to perform the work function analysis (see further below). The lattice parameters in the lateral directions are {x,y}={34.0,27.0} Å for a single adsorbed peptide while they are {x,y}={42.6,36.9} Å for a peptide nanowire. Based on these lattice parameters, the calculations subsequently carried out disclosed that four peptide molecules can fit into the supercell of a nanowire.

After molecules in each system are fully relaxed with the classical force field model, we performed *ab initio* DFT calculations to determine the induced electronic effects of the peptides onto the graphene lattice across the bio/nano interface. CMD relaxed structures have been used for self-consistent field calculations. These were carried out using the VASP code^45^ (using a CRAY XE6 machine at NERSC) and augmented plane-wave pseudopotential method^46^ with the local-density approximation using the generalized gradient correction form (GGA)^47^.  Perdew and Wang’s 1991 (PW91) gradient-corrected correlation functional has been used for exchange-correlation(Atoms, molecules, solids, and surfaces: Applications of the generalized gradient approximation for exchange and correlation: J. P. Perdew, J. A. Chevary, S. H. Vosko, K. A. Jackson, M. R. Pederson, D. J. Singh, and C. Fiolhais Phys. Rev. B 46, 6671 (1992) ). A 500 eV plane-wave kinetic energy cut-off and a single Gamma point for k-point are used. As illustrated in Fig.3, we calculated the total amount of transferred charge from the charge density in real space and the results were mapped on the surface. First, we define the charge density difference as $\Delta\rho(r)=\rho_{system}(r)-\rho_{mol}(r)-\rho_{grp}(r)$. For all quantities in this equation, the charge density is computed in the same supercell structure with the same number of grid points for a second finer FFT mesh. The quantity $\rho_{system}(r)$is the charge density of the adsorbate-graphene system. The charge density $\rho_{mol}(r)$ is the value of an isolated adsorbate peptide, which is computed with atoms fixed at their relaxed coordinates from the adsorbate-graphene calculations. The quantity of $\rho_{grp}(r)$is the charge density of isolated graphene in which atomic positions used correspond to the relaxed graphene system. We obtained 3-dimensional distribution of charge density difference of the two cases, *i.e.*, single peptide and peptide nanowire, in real space. To determine the total charge transfer based on the spatial charge density, we made use of the Bader^48^ analysis which is known to provide the total amount of transferred charge. Our analyses for both an adsorbed single peptide and a peptide nanowire (with four peptides) confirm that, in both cases, there is hole doping onto the graphene substrate. From these analyses, we found the total amount of charge transfer from graphene to peptide system to be 0.76 e^-^ for a single adsorbed molecule and a total of 2.00 e^-^ for a peptide nanowire. In other words, the total amount of average transferred charge from the graphene surface to a single peptide (averaged per peptide) decrease from a value of 0.76 e^-^ to 0.5 e^-^. In accord with the experimental evidence in hand so far, the computational results explain the difference of a single peptide versus peptide nanowire formation in their effects on electronic interaction with the graphene substrate. To assess the effects of peptides in a variety of clustering and ordering states on the surface, we performed further tests. For example, we studied the effects of two adsorbed peptides on the surface to compare its effect *versus* one isolated peptide and four peptides in a nanowire architecture. For this, we removed two of the peptides from the nanowire that originally contained four peptides and relaxed the remaining ones and calculated their total charge transfer of the system. We found that the amount of averaged transferred charge to be 0.6 e^-^per peptide molecule in the system, a values that is higher than a peptide in a nanowire architecture, but lower than a single isolated peptide on the surface. These studies imply that both the number of peptides in nanowire structure and the molecular confirmation, folding, pattern are likely to be significant in understanding the graphene-peptide electronic interactions.

Dependence of the work function of graphene with respect to peptide nanostructures could be another significant utility to evaluate the effect of the adsorbates. In general, any molecular or atomic adsorption on graphene can alter the Fermi level of graphene, and there can be large change in the work function of the hybrid system relative to the pristine graphene. It is expected that the interface dipole moment generated between the peptide and graphene will also play a significant role for the surface work function. Depending on the amount of charge transfer from graphene to the peptides (or vice versa) an electrical dipole will develop on the surface. One may calculate the work function as Φ=E_vac_-E_F_, where E_F_ is the Fermi energy while E_vac_ is the reference vacuum energy. For calculation of vacuum reference level, we applied a dipole correction (ΔV_vac_=-4πen_a_μ) in the DFT calculations. Having adsorbed molecules on graphene would generate two different vacuum levels in graphene. We designated E_vac_ as the side of graphene with adsorbed peptides. Our GGA calculations found that, while the work function value of an isolated pristine graphene is 4.2 eV, the new work functions, for single adsorbed peptide/graphene and peptide nanowire/graphene system, respectively, are 5.38 eV and 5.25 eV. Surprisingly, the effect of the nanowire architecture on graphene work function is less than that of a single adsorbed peptide. Although the total amount of transferred charge, in the case of nanowire, is higher than that of a single peptide, the question that needs to be addressed is why its effect on the work function is less.

The question why the magnitude of the change in the work function of graphene by the peptide nanowire is less compared to that of single peptide may be answered by considering molecular dipoles formed by a single peptide and a peptide nanowire. First of all, one can consider the fact that the ***z*** component of molecular dipole of an adsorbed peptide can also influence the surface work function of graphene. Therefore, one may be able to calculate the difference between the dipole moments by considering the total charge density of whole peptide nanowire/graphene system and isolated peptide/graphene system. For this, we consider that the dipole moment $\mu$in the ***z*** direction of the unit cell of a given system as $\mu=-\int\rho(z)zdz+ \sum_{k} Z_{k}ez_{k}$, where $\rho(z)$is the electron density integrated over the ***x-y*** plane, *k* is the index of ions, ***Z_k_*** is the valence electron number of ion *k*, and ***z****_k_* is the ***z*** coordinate of ion *k*. This equation gives the net dipole moment for a whole system defined in the supercell described above. Our calculations show that the total dipole moment of an isolated peptide/graphene is -25.6 D (Debye unit) and that of the peptide nanowire/graphene is -66.5 D. From these values, one can show that the ***+z*** component of dipole moment for single isolated peptide is 0.3 D while that of nanowire is 42 D. It is essential to note that when peptides form a nanowire they undergo further molecular confirmation creating a net dipole along ***+z*** direction that is different than that of a single peptide. While the positive direction of dipole moment can decrease the work function of the underlying surface, the negative value will increase it. Accordingly, we suggest that the formation of a nanowire can decrease the interaction of each peptide with the underlying graphene substrate while the work function can be decreased by effect of positive net dipole moment of the peptide nanowire. Consequently, although charge transfer from graphene to peptides increases the work function, positive molecular dipole can decrease the effect of the charge transfer and, therefore, its effect on the work function.

**S13. Effects of the substrate on the peptide organization on Graphene: Silica versus PMMA**

To investigate the effect of underlying substrate for the peptide organization, we performed studies to characterize the peptides organized on graphene supported by a PMMA-coated silicon wafer. We utilized the same condition of the peptide organization on graphene exfoliated on silicon oxide. The result is shown in Fig. S18. The morphology of peptides on single-layer graphene/PMMA is similar to the one on graphene exfoliated on silicon oxide substrate directly. The results indicate that the underlying substrate has less effect on the peptide organization on graphene surface.


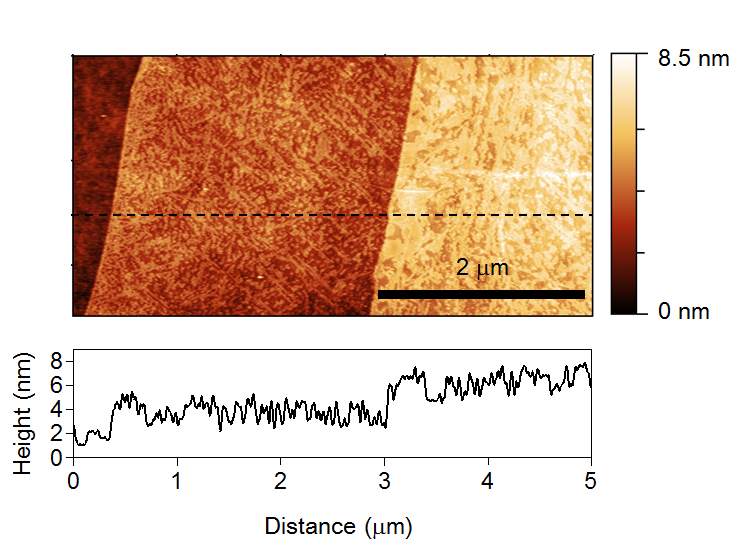


**Figure S18.** An AFM image of self-assembled GrBP5-WT peptide on a single-layer graphene, which was prepared on PMMA-coated silicon wafer, illustrates highly parallel nanowires extending from one edge of the graphene to the other. The incubation condition for the peptides was 100 nM for a period of 5 minutes. It can be noted that there is a second layer of graphene (also covered with peptides) on the right of the panel, while far to left (dark vertical region) is bare PMMA which does not display any bound peptide.

**S14. *In Situ* Observation of the Growth of Peptide Nanowires on HOPG**

The time lapse images in Fig. S19 demonstrates that, rather than forming in solution and the landing onto the substrate, the growth indeed occurs by adsorption, diffusion and assembly of the peptides on the surface.


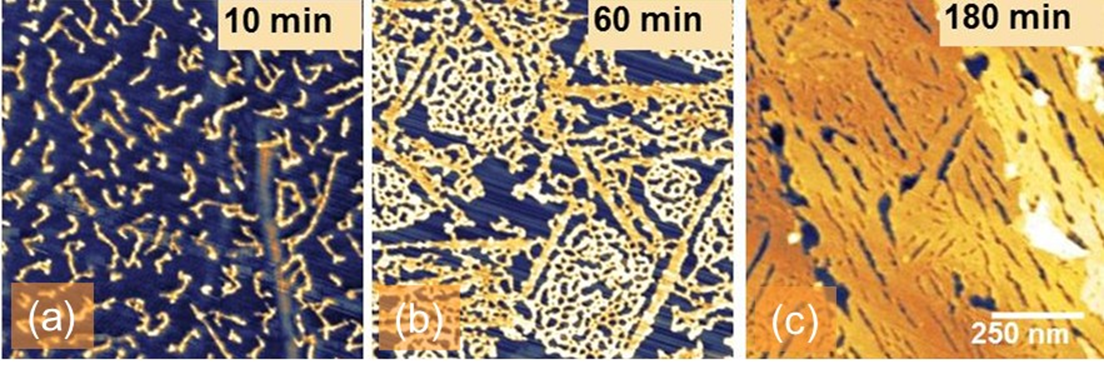


**Figure S19.** Time-lapse experiment showing peptide adsorption and growth on HOPG.^17^ The AFM images were taken after drying the samples.

The result of another experiment given in Fig. S20 displays images recorded under *in situ* AFM observations in water. Here the WT peptide was used at 5 μM concentration on HOPG. The scanning of the AFM tip had started after about 20 minutes of the experimental set up during which time the peptide had already started clustering and linearly growing on the surface. In Fig. S20, the starting time, t=0 correspond the beginning of the observations for another 8 minutes. At this starting point, the nanowire indicated by PNW already has a finite length, which then grew to be 87 nm long. From the analysis of the best fit in Fig. S20f, the linear growth rate is approximately 2.8 nm/min. The linear growth of peptides nanowires also indicates that individual peptides are assembled into the nanowires on the surface, rather than forming clusters or nanowires in the solution.


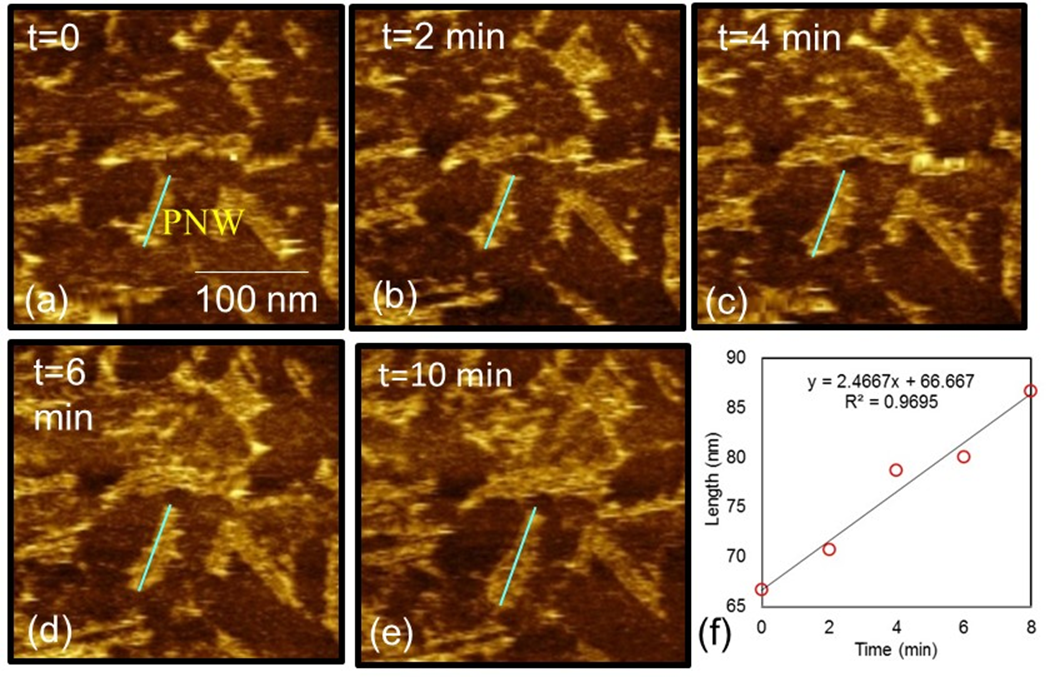


**Figure S20.** (a)-(e) In situ AFM observations of the peptide nanowire formation on HOPG. (f) Time dependence of peptide nanowire length. The green lines indicate the nanowire of interest.

**References**

1. Girit, Ç. Ö. and Zettl, A. Soldering to a single atomic layer. *Applied Physics Letters* 91, 193512 (2007).
2. Levesque, P. L., Sabri, S. S., Aguirre, C. M., Guillemette, J., Siaj, M., Desjardins, P., Szkopek, T., and Martel, R. Probing charge transfer at surfaces using graphene transistors. *Nano letters* 11, 132-137 (2010).
3. Dong, X., Fu, D., Fang, W., Shi, Y., Chen, P., and Li, L. J. Doping Single‐Layer Graphene with Aromatic Molecules. *Small* 5, 1422-1426 (2009).
4. Yan, J., Zhang, Y., Kim, P., and Pinczuk, A. Electric field effect tuning of electron-phonon coupling in graphene. *Physical review letters* 98, 166802 (2007).
5. Payne, M. C., Teter, M. P., Allan, D. C., Arias, T., and Joannopoulos, J. Iterative minimization techniques for ab initio total-energy calculations: molecular dynamics and conjugate gradients. *Reviews of Modern Physics* 64, 1045 (1992).
6. Maple, J., Hwang, M.-J., Jalkanen, K. J., Stockfisch, T. P., and Hagler, A. T. Derivation of class II force fields: V. Quantum force field for amides, peptides, and related compounds. *Journal of computational chemistry* 19, 430-458 (1998).
7. Hwang, M., Stockfisch, T., and Hagler, A. Derivation of class II force fields. 2. Derivation and characterization of a class II force field, CFF93, for the alkyl functional group and alkane molecules. *Journal of the American Chemical Society* 116, 2515-2525 (1994).
8. Plimpton, S. Fast parallel algorithms for short-range molecular dynamics. *Journal of computational physics* 117, 1-19 (1995).
9. Kresse, G. and Hafner, J. Ab initio molecular dynamics for liquid metals. *Physical Review B* 1993, 47, 558. Kresse, G., Furthmüller, J. Efficiency of ab-initio total energy calculations for metals and semiconductors using a plane-wave basis set. *Computational Materials Science* 6, 15-50 (1996).
10. Blöchl, P. E. Projector augmented-wave method. *Physical Review B* 50, 17953 (1994).
11. Perdew, J. P. and Zunger, A. Self-interaction correction to density-functional approximations for many-electron systems. *Physical Review B* 23, 5048 (1981).
12. Tang, W., Sanville, E., and Henkelman, G. A grid-based Bader analysis algorithm without lattice bias. *Journal of Physics: Condensed Matter* 21, 084204 (2009).
